# Marine and Coastal Science: Red Tide Chokehold

**DOI:** 10.1289/ehp.115-a188

**Published:** 2007-04

**Authors:** Carol Potera

The waters of the Gulf of Mexico regularly erupt with algal blooms known as Florida red tides, caused by overgrowth of the dinoflagellate *Karenia brevis*. These particular red tides release potent neurotoxins called brevetoxins. Several recently published papers help to clarify the health effects of shoreline brevetoxin aerosols, particularly for asthmatics and pregnant women. The papers were published by a team of researchers from seven institutions and funded by the NIEHS, the CDC, and the Florida Department of Health.

People who eat brevetoxin-contaminated bivalves (such as clams and oysters) develop acute gastrointestinal and neurological symptoms. When brevetoxin aerosols blow ashore, people may experience irritated eyes, coughing, and wheezing. Symptoms generally subside in healthy people once they leave the beach or enter air-conditioned buildings. Asthmatics, however, are more vulnerable to red tide aerosols, reports Lora Fleming, an epidemiologist at the University of Miami School of Medicine and Rosenstiel School of Marine and Atmospheric Sciences, with her colleagues in the January 2007 issue of *Chest*.

The team measured aerosol brevetoxin exposures and monitored symptoms in 97 asthmatics who visited Sarasota’s Siesta Beach during two active *K. brevis* blooms and three lull periods. Lung function was measured by spirometry before and after one-hour beach outings. During blooms, all participants reported an increase in symptoms (especially chest tightness) after beach exposure, and spirometry values uniformly decreased. No differences in symptoms or spirometry values were detected during lulls in *K. brevis* blooms. People with more severe asthma showed greater changes in pre- and post-beach spirometry values during red tide blooms, compared to those with mild/moderate asthma. Fleming advises sensitive people to avoid beaches when *K. brevis* is blooming and winds are blowing toward shore.

Pregnant women, too, should consider avoiding beaches during *K. brevis* blooms. Janet Benson, an inhalation toxicologist at the Lovelace Respiratory Research Institute, coordinated the first study ever of placental transfer of brevetoxins, published in the December 2006 issue of *Toxicon*. Pregnant mice received a radioactive form of brevetox-in-3, a major component of brevetoxin aerosols detected along beaches. The toxin and its by-products were identified in fetuses and uterine and placental tissues 48 hours later, as well as in the stomachs of nursing pups born to brevetoxin-exposed mothers. “The doses given pregnant mice were high and not representative of what humans are exposed to,” says Benson. Still, the results suggest that pregnant or nursing women exposed to brevetoxins may pass them to their fetuses or babies.

Animal experiments have shown that brevetoxins localize in the cerebellum, though little was known about how inhaled brevetoxins affect the brain. In the December 2006 issue of *Inhalation Toxicology*, however, the team reports that when mice inhaled brevetoxin-3 for two days, neuronal damage was observed largely in the posterior cingulated/retrosplenial cortex, but no behavioral changes occurred. The findings add to accumulating evidence that inhaled brevetoxins disperse to several body sites, though coauthor Daniel Baden, director of the Center for Marine Science at the University of North Carolina, Wilmington, says it’s too early to extrapolate to humans.

Baden, who has studied brevetoxins since 1973, says Florida red tides are occurring more frequently and often last for months. Although largely confined to the coastline along the Gulf of Mexico, *K. brevis* can travel as far north as North Carolina. Onshore concentrations of brevetoxin aerosols associated with reported respiratory symptoms range from 0.5 to 108 ng/m^3^. However, Baden says, “As far as we’re concerned, there is no dose that is low enough to not be of concern.” People have reported experiencing symptoms associated with brevetoxin exposure even when levels of the toxins were so low as to be undetectable by sophisticated monitors.

Are changes in global climate adding to the problem of Florida red tides? Scientists are studying whether changing ocean temperatures, currents, and weather patterns associated with climate change may be affecting Florida red tides. “Climate change is a concern, especially with recent blooms lasting longer, but we don’t have hard data yet,” says Barbara Kirkpatrick, manager of the Environmental Health Program at the Mote Marine Laboratory in Sarasota and a coauthor of the *Chest* paper. Her colleagues at the Mote Marine Laboratory have placed different types of sensors in several locations to monitor Florida red tide blooms and climate conditions. “It’s going to take long-term data sets to make conclusions,” Kirkpatrick says.

## Figures and Tables

**Figure:**
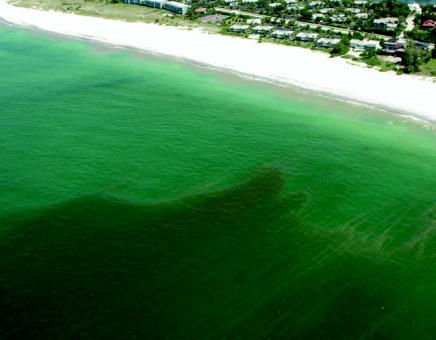
A *K. brevis* bloom approaches Coquina Beach, Florida, in 2006

